# Investigation of the Optimum Preparation of Peach Gum Polysaccharides and the In Vivo and In Vitro Therapeutic Effects on Acute Pyelonephritis

**DOI:** 10.1155/2019/2729343

**Published:** 2019-12-12

**Authors:** Feifei Zhang, Jie Bai, Yao Zheng, Shuai Liang, Lei Lei, Xin Deng, Weijun Li, Peng Liu, Guangzhong Yang, Yongshen Ren

**Affiliations:** School of Pharmaceutical Science, South-Central University for Nationalities, Wuhan 430074, China

## Abstract

**Background:**

Acute pyelonephritis (APN), known as stranguria in traditional Chinese medicine, is commonly treated with antibiotics. However, the rise in antibiotic resistance and the high rates of recurrence of APN make its treatment complicated, thus the development of alternative therapies is critical. Peach gum has long been recognized by traditional Chinese medicine as a food with medicinal value of relieving stranguria, but whether and how its primary constituent peach gum polysaccharides (PGPs) contribute to the diuretic function is still not clear.

**Purpose:**

The aim of this study was to investigate the optimum extraction process of PGPs and to evaluate its therapeutic effect on APN rats and to discover the underlying mechanism.

**Methods:**

In this study, surface design optimization was adopted to optimize the preparation of PGPs and HPLC and FT-IR spectra were used to evaluate the quality of PGPs; APN model rat was established by the *Escherichia coli* urinary tract infection method; the therapeutic effect and mechanism of PGPs on APN were determined by the visceral index, biochemical indicators, pathological section of the APN rat, and diuretic activity on mice and antibacterial activity in vitro.

**Results:**

Compared with an untreated APN group, the results showed that treatment with PGPs increased the APN-induced attenuation of secretory immunoglobulin A (sIgA) and creatinine clearance and decreased the APN-induced enhancement of the number of white blood cell (WBC), neutrophil counts (NC), bacteria load of the kidneys, kidney index, serum creatinine, urine volume, blood urea nitrogen (BUN), and interleukin-2 (IL-2) levels. The mechanism underlying these effects was further elucidated through in vitro experiments of the antibacterial and antiadhesion effects of PGPs.

**Conclusion:**

Due to the good therapeutic effects and advantages of PGPs, it could be considered as an alternative medicine to treat APN.

## 1. Introduction

Urinary tract infections (UTIs) are some of the most common bacterial infections, affecting 150 million people worldwide each year [1]. The predominant cause of UTIs in humans is uropathogenic *Escherichia coli* (UPEC), which causes 75–95% of UTIs. Women are more highly predisposed to UTIs than men, primarily due to anatomic differences in the urogenital tract [2].

UTIs are usually ascending in nature, beginning with bacterial colonization and inflammation of the urinary bladder (cystitis) [3, 4]. This then rises up to the kidneys via the ureters, resulting in inflammation of the renal pelvis and parenchyma, known as acute pyelonephritis (APN).

APN is accompanied by fever, frequent voiding of small volumes of urine, and flank pain and requires immediate medical attention. Uncontrolled APN may lead to tubular atrophy, interstitial inflammation, and interstitial fibrosis [5]. Antibiotic therapy is the most common therapeutic method for APN [6]. However, there remain significant challenges in clinical practice due to the prevalence of APN, its high recurrence rate, and the worldwide increase in antibiotic resistance [7].

Due to the challenges described above, alternative treatments should be encouraged. To this end, traditional Chinese medicine (TCM) has great potential. In TCM, UTIs are regarded as stranguria syndrome, which encompasses urolithiasis, bloody stranguria, pyretic stranguria, and chyluria stranguria, among others; APN is the most similar to pyretic stranguria. Treatment of APN with TCM can be divided into two stages [8]: the acute stage and the nonacute stage. In the acute stage, the main treatments include heat-clearing, detoxifying, symptom relief, and reduction in the discomfort of the urethra. Antimicrobial medicines should also be added to consolidate the effects of the TCM treatments and reduce the recurrence rate.

Peach gum is a popular food with medicinal value. 1500 years ago, the *Tang Materia Medica* (*Tang Ben Cao*) recorded that people use peach gum as a kind of medicine. In TCM, peach gum is considered to have diuretic properties and can relieve stranguria and pain [9]; thus, the properties of peach gum suggest that it is well suited to the treatment of APN. Modern research has found that the main components of peach gum are polysaccharides [10]; extraction of polysaccharides from peach gum can overcome the problems that limit the clinical application of peach gum, such as its poor solubility in water [11]. In recent studies, peach gum polysaccharides (PGPs) have been found to regulate blood sugar levels [12], have antiaging [13], antioxidant, and antimicrobial [14] properties, and can boost immunity [15]. However, to date, no studies have shown the therapeutic effect of PGPs on APN. To this end, the current study demonstrates the therapeutic effect of PGPs on APN through animal experiments.

In this study, using the water extraction method, alcohol precipitation was used to extract PGPs; this method was combined with response surface methodology (RSM) to optimize the maximum yield of PGPs. Then, we performed in vivo and in vitro studies to demonstrate the therapeutic effects of PGPs on APN and to clarify the possible mechanisms.

## 2. Materials and Methods

### 2.1. Chemicals and Reagents

Peach gum (lot no: 20160415; Suizhou, China) was purchased from the Bozhou Special Market for Chinese Materia Medica and was identified as the gelatinous secretion of *Prunus persica* (L.) Batsch. The peach gum was ground into powder and passed through a 0.2 mm sieve prior to extraction of the polysaccharides. Dichloromethane (lot no: 20170212) was purchased from Tianjin Fuyu Fine Chemical Co., Ltd. D-(+)-xylose (lot no: 20160406) and 1-butanol (lot no: 20171109) were purchased from x. (Shanghai, China). Yeast extract (20259606–02) and tryptone (1765365) were obtained from OXOID Ltd. (made in England). Agar (EZ2811E322) was purchased from Guangzhou Saiguo Biotech Co., Ltd. (Guangzhou, China). Levofloxacin hydrochloride capsules (lot no: 20170605) were manufactured by Jilin Wantong Pharmacy Group Co., Ltd. (Jilin, China). Furosemide tablets (1705085) were produced by Jiangsu Asia Pharmaceutical Co., Ltd. (Jiangsu, China). The Rat IL-2, sIgA, CRP Elisa kit (lot no. 20180510), creatinine assay kit (lot no. 20180428), BUN assay kit (lot no: 20180604), ALT/GPT (lot no. 20180329), and AST/GOT assay kit (lot no: 20180329) were purchased from the Nanjing Jiancheng Bioengineering Institute. All other chemicals used were analytical grade and were purchased from local suppliers.

### 2.2. Optimization of PGPs Extraction by Response Surface Methodology (RSM)

#### 2.2.1. Extraction of PGPs

Water extraction with the alcohol precipitation method was adopted to extract PGPs [16]. Briefly, the peach gum powder was soaked in proper quantities of distilled water and boiled at 100°C for a certain period of time. Then, the extract was filtered and concentrated. Subsequently, the protein was removed by the same volume of the sevage reagent (the ratio of dichloromethane to 1-butanol (v/v) is 5 : 1) and the upper aqueous layer was kept for the next step. The reserved aqueous layer was bleached with a quarter volume H_2_O_2_ (content 30%) for 0.5 hour and then incubated overnight with 4 volumes of ethanol. Thereafter, the precipitated polysaccharides were centrifuged and collected. Here, the extractive water consumption, decocting time, and precipitative ethanol concentration were evaluated using the single factor design and response surface methodology.

#### 2.2.2. Single-Factor Investigation

The leading factors such as the decocting time, ratio of water to raw material, and concentration of precipitation ethanol significantly affect the extraction efficiency of the above-described extraction process. In each experiment, all other two factors were kept constant and the level of one factor was changed to investigate the optimal conditions for extraction yield.

#### 2.2.3. RSM Investigation

Based on the results of the preliminary single-factor investigation, the effects of decocting time (*X*_1_), ratio of water to raw material (*X*_2_), and ethanol concentration (*X*_3_) were optimized by RSM. Design-Expert software was used to observe the response surface graphs. The parameters and their levels are presented in Table 1.

RSM provides an empirical relationship between the response function and the independent variables. The quadratic response model is based on all linear terms, square terms, and linear interaction terms to investigate the model adequacies:

(1) $$\begin{array}{c} Y=\beta_{0}+\overset{3}{\underset{i=1}{\sum }}\beta_{i}X_{i}+\overset{3}{\underset{i=1}{\sum }}\beta_{ii}X_{i}^{2}+\overset{3}{\underset{i=1}{\sum }}\overset{3}{\underset{j=i+1}{\sum }}\beta ijX_{i}X_{j}. \end{array}$$

Analysis of variance (ANOVA) was selected to test the statistical significance of the regression coefficients [17].

#### 2.2.4. Determination of PGP Content

The reducing sugar content and total sugar were analyzed by the 3, 5-dinitrosalicylic (DNS) colorimetric method using D-(+)-xylose as the standard. Li et al. [18] described the method of total sugar content determination by DNS. The absorbance was measured at 500 nm, and the total concentration of sugar was calculated using equation (3) based on a standard curve obtained with D-(+)-xylose. The response surface diagram was constructed by taking the weighted results obtained after the homogenization of total sugar and the extraction rate as the response value (weight).

### 2.3. FT-IR Spectral Analysis and Content Determination of PGPs

#### 2.3.1. High-Performance Liquid Chromatography (HPLC) and Content Determination of PGPs

A certain concentration of a mixed standard solution was prepared; the monosaccharide standards included D-mannose, D-rhamnose, D-glucose, D-galactose, D-xylose, and L-arabinose. Wan et al. [19]. described the experimental process of hydrolysis of polysaccharide samples and derivatization of monosaccharides. Based on this previous report, the monosaccharide composition was measured by acid hydrolysis prior to HPLC. Briefly, the hydrolysate of the polysaccharide fractions and mixed standard solution were heated with 1-phenyl-3-methyl-5-pyrazolone (PMP) at 70°C for 60 min. The final solutions were analyzed on a Dionex Ultimate 3000 HPLC instrument using a C_18_ column (250 mm × 4.6 mm, 5 *μ*m; Hypersil BDS-C_18_ column) at 30°C with a flow rate of 1 mL/min. The injection volume for all the samples tested in this study was 10 *µ*L with the wavelength for UV detection at 254 nm. Monosaccharide compositions were identified by comparison with the retention times of the monosaccharide standards.

#### 2.3.2. FT-IR Spectra of PGPs

The FT-IR spectra of PGPs were obtained using a Fourier-transform infrared spectrophotometer. The dried sample (2 mg) was ground together with KBr powder. Then, this was pressed into 1 mm pellets for Fourier-transform infrared spectrophotometer (FT-IR) measurement in the infrared region of 4000–400 cm^−1^ via the pressed disc method.

### 2.4. Experimental Design of APN

#### 2.4.1. Animals

Experiments were performed on 6–8 week-old female Sprague Dawley (SD) rats (SCXK (Liao) 2015–0001) weighing 200 ± 20 g. The animals were kept in a controlled environment at a temperature of 24–26°C, humidity of 55–60%, and a 12 h light/dark cycle with unlimited access to water and food. All experimental animal processing programs were performed strictly in accordance with the Institute of Experimental Animal Management of South-Central University for Nationalities and the Guide for the Care and Use of Laboratory Animals issued by the National Institutes of Health in 1996. The protocols used in this study were in accordance with the Regulations of Experimental Animal Administration issued by the Ministry of Science and Technology of the People's Republic of China (http://www.most.gov.cn).

#### 2.4.2. Bacteria

The *Escherichia coli* (*E. coli*) strain (CMCC 44817) was cultured in the Luria broth (LB; 10 g/liter tryptone, 5 g/liter yeast extract, and 0.5 g/liter NaCl) at 30°C.

Before infection, a single colony of bacteria was inoculated into the Luria-Bertani (LB) broth and shaked at 30 °C till the bacterial growth reached the plateau stage. The solution contains approximately 5 × 10^7^ organisms/mL.

#### 2.4.3. Experimental Groups

Animals were randomly allocated into six groups, 10 rats each. The normal control group (normal) consisted of healthy rats. The rats in the APN group had APN and did not receive any treatment. APN was induced by the injection of *E. coli* to the remaining rats, as described below. In the treatment groups, treatments began 12 hours after bacterial inoculation. In the levofloxacin hydrochloride capsule (LHC) group, the rats received LHC (positive drug) 30 mg/kg daily by gavage. In the low-dose PGP group, rats received PGP 2 g/kg by gavage. The rats in the middle-dose PGP group received daily intragastric PGP at a dose of 4 g/kg. In the high-dose PGP group, the rats received 6 g/kg daily by gavage.

#### 2.4.4. Experimental Infection

Before the operation, the rats were fasted and deprived of water for 12 h. All animals except the normal group were anesthetized by an intraperitoneal injection of urethane at 1.2 g/kg. Each anesthetized rat was infected with 5 × 10^7^ organisms per milliliter of *E. coli* nutrient solution; an epidural catheter (0.7 mm outer diameter) was used for transurethral delivery of about 0.6 mL of inoculum into the bladder. After inoculation, the catheter was removed and the outer orifice of the urethra was sealed with artery occlusion to avoid leakage and reflux. Eight hours later, the artery occlusion was removed by gentle massage. The animals were monitored regularly for any discomfort, injury, or inflammation due to the procedure [20–22].

#### 2.4.5. Sampling and Analysis of the Material

On the 6th and 12th day after experimental infection, the urine samples were collected using a metabolic cage for determination of biochemical parameters and measurement of 24 h urine volume.

On the 14th day, all animals were anesthetized via intraperitoneal injection of urethane solution (1.2 g/kg); the rats were fasted before the operation. Blood samples were collected into two vacuum blood collection tubes; a 5 mL blood sample taken from the abdominal aorta of each rat was placed into a tube without an anticoagulant, and a 1 mL blood sample was placed in a tube with EDTA.K_2_. The blood samples with an anticoagulant were used for a hematological study. After sanding for 30 min, the blood samples without an anticoagulant were centrifuged for 15 min at 3500 rpm. Then, the separated serum was stored in Eppendorf tubes at −80°C in a refrigerator for later measurement.

Meanwhile, the rats were sacrificed by cervical dislocation. Then, the kidneys, bladder, liver, and spleen were removed, decapsulated, washed with saline, and weighed. Half of the left kidney and a piece of the liver were fixed in 10% formalin solution for histopathological analysis. The right kidney and another half of the left kidney were stored at −80°C for later use. Other tissues were also stored at −80°C.

#### 2.4.6. Measurement of Relative Organ Weight of Kidney, Bladder, and Spleen

Organ weight was recorded. The relative organ weight (ROW) of the kidney and bladder were calculated using the following equation:

(2) $$\begin{array}{c} \text{ROW}=\frac{\text{Absolute organ weight}\left(g\right)}{\text{Body weight of rat on the day of sacrifice}\left(g\right)}\times 100. \end{array}$$

#### 2.4.7. Hematological Analysis

The anticoagulated blood samples were used for a hematological study which involved the measurement of white blood cell (WBC) count and neutrophil counts (NC). The serum was used for a biochemical study which involved measurement of creatinine activity, urea nitrogen, aspartate aminotransferase (AST), alanine aminotransferase (ALT), CRP (ELISA), and IL-2 (ELISA). All assays were performed using commercial kits obtained from the Nanjing Jiancheng Bioengineering Institute.

#### 2.4.8. Measurement of Urine Parameters

Creatinine level, NAG, and sIgA (ELISA) were measured from the urine samples. All parameters were analyzed using commercial kits obtained from the Nanjing Jiancheng Bioengineering Institute.

#### 2.4.9. Measurement of Microbial Counts in Kidney

For the microbiology study, the sample (half of the left kidney, 0.1 g) was homogenized in 0.9 mL of sterile saline. Then, 100 *μ*L kidney homogenate was placed on the LB solid medium. The number of colony-forming unit (CFU) of *E. coli* in each kidney was determined after incubation for 18 h at 7°C. When no CFU was detected on the agar, the kidney was considered sterile [5].

#### 2.4.10. Hemagglutination Assay

Tang et al. [23] described the method of preparing red blood cells in rabbits. According to this previous report, the prepared red blood cells were diluted in physiological saline (20% v/v), divided into 2 mL/tubes, centrifuged, and stored at 4°C for 12 h before use [24]. The prepared red blood cells were used within 72 hours.

Antiadhesion activity was tested by measuring the ability of urine samples to suppress agglutination of rabbit red blood cells, following incubation with *E. coli*. The *E. coli* strain used in these assays was isolated from the urine of the normal and APN model rats. The bacteria isolated from the rats were subcultured on colonization factor agar media at 37°C in order to enhance the expression of P fimbriae. Kaspar et al. [25]. described the experimental process of the hemagglutination assay. After preparation of the bacterial suspensions (5 × 10^8^ bacteria mL^−1^), 10 *μ*L of bacterial suspension was incubated with a 30 *μ*L urine sample on a 24-well polystyrene plate and placed on a shaker for 10 min at ambient temperature. Rabbit red blood cells were diluted in PBS (3% v/v), and 10 *μ*L was added into the urine-bacterial suspensions. The samples were incubated at 21°C for 20 min on a shaker and evaluated microscopically for their ability to prevent agglutination. Results are reported on an ordinal scale with antiadhesion activity as follows: 0 = 0% adhesion inhibition; 1 = 50% inhibition; and 2 = 100% inhibition. The percentages are approximate, and the results are qualitative, with activity being present or not present.

#### 2.4.11. Histopathological Analysis of Kidney and Liver Tissues

The entire kidney tissue section (5 mm) and a piece of liver were fixed by immersion at room temperature in 10% formalin solution. For histopathological examinations, paraffin-embedded kidney tissue sections were stained with hematoxylin eosin (HE) and were then examined and photographed. A light microscope was used to observe structural abnormalities.

### 2.5. Experimental Design of Diuretic Activity

#### 2.5.1. Animals

Thirty female mice (SCXK (E) 2015–0018) (18–22 g) were randomly divided into five groups (*n* = 5). Each animal was placed in an individual metabolic cage 24 h prior to commencement of the experiment for adaptation. In a parallel experiment, mice were fasted overnight with free access to water and then subjected to the treatment described below. All experimental animal processing programs were performed strictly in accordance with the Institute of Experimental Animal Management of South-Central University for Nationalities.

#### 2.5.2. Diuretic Activity Evaluation of PGPs

Before treatment, all animals were administered physiological saline (i.p.) at an oral dose of 50 mL/kg body weight (BW) to impose a uniform water load. 30 min later, the mice were treated orally in the following manner: the normal control group was given normal saline 15 ml/kg BW; the furosemide group received 30 mg/kg BW; the LHC group received 30 mg/kg; and the low-dose, middle-dose, and high-dose groups were given PGP at a dose of 2 g/kg, 4 g/kg, and 6 g/kg, respectively. Immediately after treatment, the mice were put into separate metabolic cages and the volumes of urine were measured hourly for four hours over a three-day period [26].

### 2.6. Antibacterial Activity of PGPs

#### 2.6.1. Materials

*E. coli* (CMCC 44817), *Staphylococcus aureus* (*S. aureus*) (ATCC 29213), *Micrococcus luteus* (*M. luteus*) (CMCC 28001), and *Aerobacter aerogenes* (*A. areogenes*) (ATCC 49701) were obtained from the College of Life Sciences of South-Central University for Nationalities. Oxford cups were purchased from Jingke Apparatus and Equipment Cooperation Ltd. (Beijing, China).

#### 2.6.2. Agar Well Diffusion Method of Antibacterial Activity and Measurement of Minimum Inhibitory Concentration (MIC)

All experimental equipment was sterilized before the experiments. Each bacterium was activated in LB liquid medium for 12 h at 37°C. Culture suspensions (100 *μ*L) were spread on LB agar. PGP was dissolved in sterile water to different concentrations (500 mg/mL, 250 mg/mL, 125 mg/mL, 100 mg/mL, and 75 mg/mL, respectively). The final concentrations of each bacterium were about 10^7^ CFU/mL, as determined by the colony-counting method. Four Oxford cups were placed on a solid culture plate (*D* = 7.8 mm). 200 *μ*L of different concentrations of PGPs and sterile water (as control) were dropped into four holes. Thereafter, the culture plates were diffused for 10 h at 4°C and then incubated at 37°C for 24 h. After incubation, the clear zones were regarded as inhibitory zones and recorded in mm. The results are shown in Table 2. MIC was determined using the abovementioned method, and tests were conducted following the determination of the zone of inhibition mentioned above [27].

### 2.7. Statistical Analysis

All data were analyzed with GraphPad Prism version 5.0. Data were expressed as mean ± SD. *t*-tests were used to compare the control and treatment samples. A *P* value less than 0.05 (*P* < 0.05) was considered statistically significant.

## 3. Results

### 3.1. Single-Factor Experiments for Extraction of PGPs

**Figure 1:**
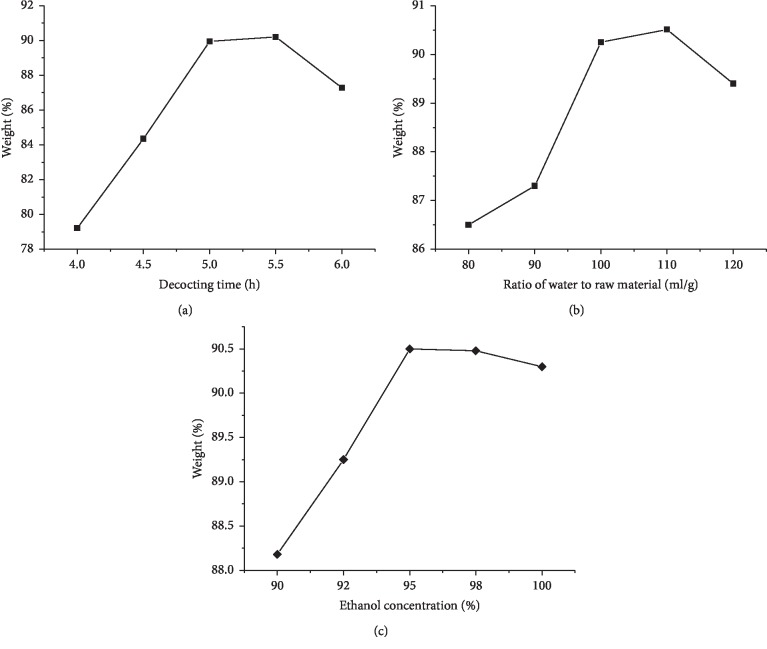
Effects of decocting time (a), ratio of water to raw material (b), and ethanol concentration (c) on the total sugar and extraction yield of PGPs (weight).

The effects of the extraction parameters on the total sugar and extraction yield (weight) of PGPs are shown in Figure 1.

### 3.2. Influence of Decocting Time on the Yield of PGPs

The decocting time was set at 4 h, 4.5 h, 5 h, 5.5 h, and 6 h, respectively. The other two factors (ratio of water to raw material and concentration of precipitation ethanol) remained constant: the ratio of water to peach gum powder was 100 : 1 (m/m) and the ethanol concentration was 95%. As presented in Figure 1(a), an extraction time of 5 h was chosen for use in the following experiments.

### 3.3. Influence of Ratio of Water to Raw Material on the Yield of PGPs

The ratio of water to raw material is an important parameter in the extraction process of water-soluble polysaccharides. To examine its effects, decocting time was set to 5 h, ethanol concentration was 95%, and the following water to peach gum powder ratios were investigated: 80 : 1, 90 : 1, 100 : 1, 110 : 1, and 120 : 1. As shown in Figure 1(b), a ratio of water to peach gum powder of 100 : 1 was selected for use in the following experiments.

### 3.4. Influence of Ethanol Concentration on the Yield of PGPs

Concentrations of ethanol ranging from 90% to 100% were investigated; the ratio of water to peach gum powder was set at 100 : 1, and the decocting time was set at 5 h.  Figure 1(c) shows that an ethanol concentration of 95% was suitable for use in the following experiments.

### 3.5. Content of Total Sugar

The linear relationship between polysaccharide concentration and OD_500_ can be expressed as follows:

(3) $$\begin{array}{c} Y=0.0019X+0.1386,\;R^{2}=0.9991. \end{array}$$

As shown in Table 2, weighting of the xylose content and extraction yield was regarded as the basis for optimization of the extraction process.

### 3.6. Optimization of the Extraction Yield by RSM

In the RSM process, the fitted model that was used for the extraction yield of PGPs (%) was applied to predict the relationship between the independent variables and the dependent variable; this model can be expressed as follows:

(4) $$\begin{array}{c} Y=90.69+4.10X_{1}+1.43X_{2}+1.24X_{3}-0.18X_{1}X_{2}-0.48X_{1}X_{3}-0.11X_{2}X_{3}-7.35X_{1}^{2}-2.25X_{2}^{2}-1.27X_{3}^{2}. \end{array}$$

As shown in Table 3, a model *F* value of 98.56 and a low probability *P* value (<0.0001) indicate that the model was highly significant. The  *R*_Adj_^2^ value indicates that 98.21% of the total variation in the yield of PGPs was attributed to the independent variables, and only about 1.79% of the total variation could not be explained by the model. Based on the evidence mentioned above, it can be concluded that the model equation is adequate for predicting extraction yield of PGPs (weight) in this study. The primary and secondary factors influencing the yield of PGPs were extraction time > extraction temperature > ethanol concentration.

Based on the above indexes, the regression equation was analyzed and the optimal extraction conditions for PGPs were obtained as follows: decocting time 5.13 h, ratio of water to raw material 102.67 mL/g, and ethanol concentration 95.67%. The maximum predicted extraction yield of PGPs was 91.3873% (weight). In order to save time and money, in practice, the actual extraction conditions were modified slightly: ratio of water to raw material 100 mL/g, decocting time 5 h, and ethanol concentration 95%.

### 3.7. Composition Characteristics of PGPs

#### 3.7.1. HPLC Chromatograms and Content Determination of PGPs

**Figure 2:**
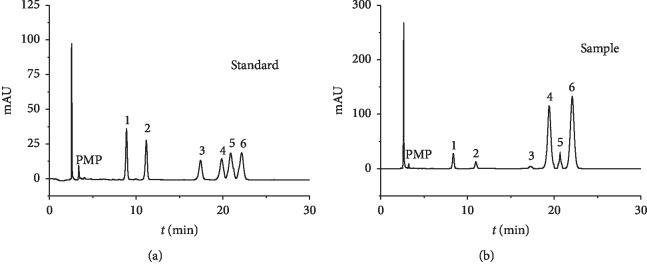
HPLC chromatograms of monosaccharide derivatives of hydrolyzed polysaccharides from PGPs by PMP derivatization.

As compared with PMP-labeled standard monosaccharides, PGPs were composed of mannose, rhamnose, glucose, galactose, xylose, and arabinose with a content of 4.10 : 2.15 : 1.74 : 34.52 : 7.56 : 49.93, respectively. The monosaccharide compositions of PGPs and the quantitative results are shown in Table 4 and Figure 2.

#### 3.7.2. Infrared Spectrum Analysis

The FT-IR spectra of PGPs peaked at around 3398.57 cm^−1^; this can be assigned to the stretching vibrations of hydrogen-bonded OH groups. Peaks at ∼2933.73 cm^−1^ (unsymmetrical stretching vibration), ∼1720 cm^−1^ (special absorbance peaks of aldehyde in PGPs), ∼1419.61 cm^−1^ (symmetrical deformation of -CH_3_ and -CH_2_), and ∼1050 cm^−1^ are due to the stretching vibration of the C-O-C bond in the glucose circle.

### 3.8. Results of APN Experiment

#### 3.8.1. General Condition of Rats

In the normal group, the rats were lively with thick and shiny hair. They had normal food and water intake, brownish black and well-formed stools, and normal urination during the experiment.

The first day after modeling, the rats in the APN group began to ingest less food and water and had formed but slightly loose stools. On the 3^rd^ day after modeling, the rats exhibited clustering, arching of the back, curling, and ingested less food and water. From the 5^th^ day after modeling, the rats gradually became more active and increased food and water intake. As of the 6^th^ day after modeling, 24 h urine output was increased (Table 5). On the 2^nd^ day after modeling, two rats died intermittently. These deaths might have been caused by the modeling, adverse reactions to the bacteria, or individual differences.

In the LHC group, the rats were weak from the first day after modeling; they ingested less food and water, and their stools were loose, smelly, and darker in color. From the 3^rd^ day after modeling, the rats started to drink water independently. On the 3^rd^ day after modeling, one rat died; this may be due to the modeling or individual differences.

In the low-dose PGP group, from the day after modeling, the rats exhibited a poor mental state and ingested less food and water. They exhibited clustering, curling, and arching of the back. From the 5^th^ day after modeling, their mental state had deteriorated further than the middle-dose PGP group, but their stools were the same as before. Three rats died during the experiment; this may be caused by individual differences or adverse reactions to the bacteria.

In the middle-dose PGP group, as of the first day after modeling, the rats ingested less food and water and their stools were formed but loose. During the experiment, their stools were not the same as before, which became more soft and which may be caused by the concentration of PGP solution. From the 3^rd^ day after modeling, the rats returned to their normal condition. One rat died during the experiment; this may be caused by individual differences.

In the high-dose PGP group, from the first day after modeling, the rats ingested less food and water and were very weak; their stools tended to look like a string of beads, and the anus was often dirty. This phenomenon was probably due to the high concentration and high viscosity of the PGPs. During the experiment, two rats died; this might be due to adverse reactions to the bacteria.

In summary, the high-dose and middle-dose treatment groups were better than the APN group with respect to general condition. Great differences in urine volume and mental state were evident between the two groups.

#### 3.8.2. 24 h Urine Volume

The day before the rats were sacrificed, the rats in the administration groups presented with lower urine output than the APN group. The urine outputs of the APN group and the low-dose PGP group were significantly increased compared with that of the normal group (*P* < 0.0001). Compared with the APN group, the urine volume of the high-dose PGP group was significantly decreased (*P* < 0.01). The urine outputs of the middle-dose PGP group and the LHC group were significantly lower than that of the APN group (*P* < 0.05). No significant difference was seen between the high-dose PGP group and the normal group (*P* > 0.05) (Table 5).

#### 3.8.3. Comparisons of Relative Organ Weight and Indices

Relative organ weights and indices for each group are shown in Table 6.

The kidney weights of the treatment groups were significantly lower than those of the APN group (*P* < 0.01). The kidney weights of the APN group were significantly higher than those of the normal group (*P* < 0.01). Compared with the APN group, the LHC, low-dose, and high-dose groups have significantly decreased the weights of the kidney (*P* < 0.01 and *P* < 0.05, respectively). Meanwhile, the kidney index of the APN group was significantly increased compared with that of the normal group (*P* < 0.001). The kidney indices of the LHC group and the low-dose PGP group were significantly decreased compared with that of the APN group (*P* < 0.01). Furthermore, the kidney index of the high-dose PGP group was significantly lower than that of the APN group (*P* < 0.001).

The bladder weights of the APN group and all of the treatment groups, except the high-dose group, were significantly higher than that of the normal group (*P* < 0.001). The bladder weights of the LHC group and the high-dose group were significantly decreased compared with that of the APN group (*P* < 0.05 and *P* < 0.01, respectively). Meanwhile, the bladder indices of the APN group, LHC group, low-dose PGP group, and middle-dose PGP group were significantly higher than that of the normal group (*P* < 0.001). The bladder indices of the LHC group and the high-dose PGP group were significantly lower than that of the APN group (*P* < 0.05 and *P* < 0.01, respectively).

The spleen weight of the APN group was significantly higher than that of the normal group. However, compared with the APN group, the spleen weights of the low-dose and middle-dose PGP groups were significantly decreased. Meanwhile, the spleen indices of the APN and the LHC groups were significantly lower than that of the normal group (*P* < 0.05). There were no significant differences between the APN and LHC groups. However, compared with the APN group, the spleen indices of the low-dose and middle-dose PGP groups were significantly increased (*P* < 0.01).

#### 3.8.4. Levels of BUN, SCr, UCr, and ClCr

Changes in the levels of blood urea nitrogen (BUN), serum creatinine (SCr), urine creatinine (UCr), and creatinine clearance (ClCr) are shown in Table 7.

In the control group, BUN levels were in the normal range, while levels were increased in the APN group; this difference was statistically significant (*P* < 0.05). BUN levels in low-dose, middle-dose, and high-dose groups were significantly decreased compared with that of the APN group (*P* < 0.001).

SCr levels were in the normal range in the control group, but were significantly increased in the APN group (*P* < 0.05). SCr levels in the LHC and low-dose PGP groups were significantly decreased compared with that of the APN group (*P* < 0.05). In the high-dose PGP group, SCr levels were significantly decreased compared with the APN group (*P* < 0.01).

UCr levels in the treatment groups were significantly decreased compared with that of the APN group. The UCr concentration of the APN group was significantly higher than that of the control group (*P* < 0.01). There was a significant difference in UCr concentration between the middle-dose PGP group and the APN group (*P* < 0.001). Compared with the model group, the UCr level of the high-dose PGP group was significantly decreased.

ClCr levels of the treatment groups were significantly decreased compared with that of the APN group. The ClCr level of the APN group was significantly lower than that of the control group. Compared with the APN group, the LHC, low-dose PGP, and high-dose PGP groups were significantly increased. There was no significant difference between the control group and the low-dose PGP group.

#### 3.8.5. WBC Count and Neutrophil Count

**Figure 3:**
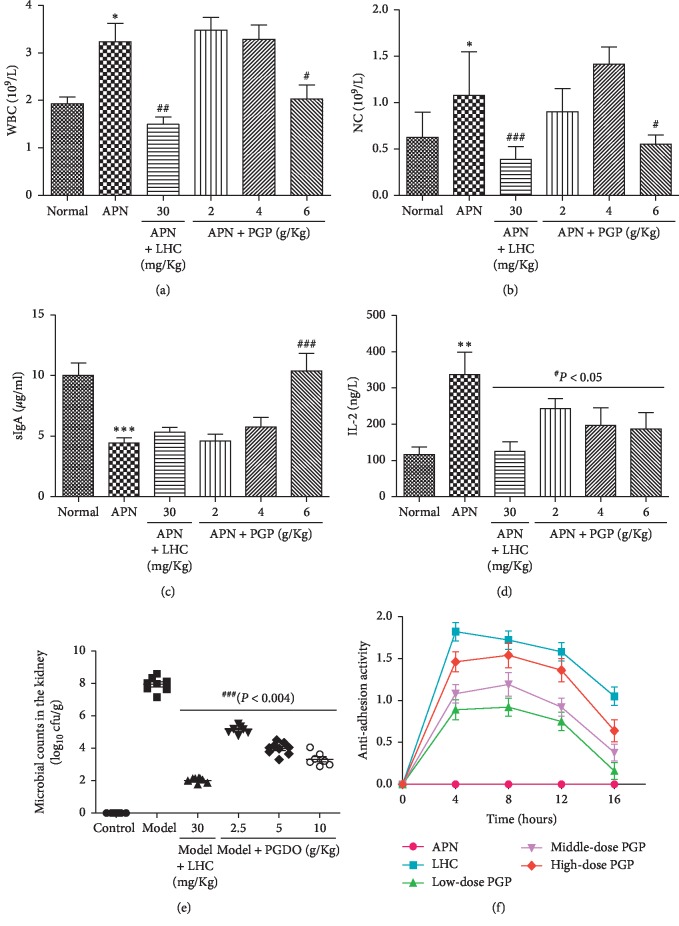
Levels of 6 kinds of biochemical indicators: (a) white blood cell counts, (b) neutrophil counts, (c) sIgA concentration in urine, (d) IL-2 concentration, (e) microbial counts in the kidney, and (f) antiadhesion activity. ^*∗*^*P* < 0.05 compared with the control group; ^*∗∗*^*P* < 0.01 compared with the control group; ^#^*P* < 0.05 compared with the APN group; ^##^*P* < 0.01 compared with the APN group; ^###^*P* < 0.001 compared with the APN group.

WBC counts were significantly increased in the infected groups. The WBC count of the LHC group was significantly lower than that of the APN group (*P* < 0.01). The WBC count of the high-dose PGP group was significantly decreased compared with that of the APN group (*P* < 0.05), and with increasing dosage, the WBC count of the PGP groups showed a downward trend (Figure 3(a)).

The neutrophil count (NC) of the APN group was markedly increased compared with that of the normal group (*P* < 0.05). Compared with the APN group, the NC of the LHC group was significantly decreased (*P* < 0.001). The NC was significantly decreased in the high-dose PGP group compared with the APN group (*P* < 0.05) (Figure 3(b)).

#### 3.8.6. Results of sIgA, CRP, and IL-2 Test

sIgA concentrations in urine were significantly increased in the treatment groups as compared with the APN group. With increasing PGP dosage, the sIgA concentrations of the PGP groups increased. Compared with the normal group, the sIgA concentration of the APN group was markedly decreased (*P* < 0.01). The concentration of sIgA in the LHC group was significantly decreased compared with the APN group. Furthermore, high-dose PGP treatment significantly increased the concentration of sIgA (*P* < 0.01). The results are shown in Figure 3(c).

According to the results in Figure 3(d), the concentrations of IL-2 in the treatment groups were significantly decreased compared with that of the APN group. In the PGP groups, the IL-2 concentration showed a downward trend with increasing dosage. The IL-2 concentration of the APN group was significantly higher than that of the control group (*P* < 0.01). The LHC, middle-dose, and high-dose PGP treatment groups showed a significant decline in IL-2 concentration compared with the APN group (*P* < 0.05).

According to the results in Table 7, the concentrations of CRP in the treatment groups were significantly decreased compared with that of the APN group. The CRP concentration of the APN group was significantly higher than that of the control group (*P* < 0.01). The LHC, low-dose, middle-dose, and high-dose PGP treatment groups showed a significant decline in CRP concentration compared with the APN group (*P* < 0.05). With increasing of PGP dosage, the CRP concentrations of the PGP groups decreased.

#### 3.8.7. Microbial Counts in Kidney and Hemagglutination Assay

The cfu/mL of *E. coli* in the kidney homogenate of the APN group was increased significantly compared with the normal group (sterile kidney). The microbial counts in the kidney homogenates of treatment groups were decreased significantly compared with the APN group. The microbial count in the kidney of the LHC group was significantly lower than those of the PGP groups. The PGP groups tended to show a dose-dependent decrease in microbial counts (Figure 3(e)).

The antiadhesion activity results showed that at the 4^th^ hour, the LHC and PGP groups were all on the rise, and the LHC group tended to rise faster than the PGP groups. Undoubtedly, the best antiadhesion effect was in the LHC group, even though at the 8^th^ hour it exhibited a downward trend. However, among the PGP groups, the high-dose PGP group had the best effect, and from the 4^th^ to the 8^th^ hour it exhibited an upward trend. The PGP groups also exhibited a dose-dependent manner (Figure 3(f)).

#### 3.8.8. Level of AST and ALT

**Figure 4:**
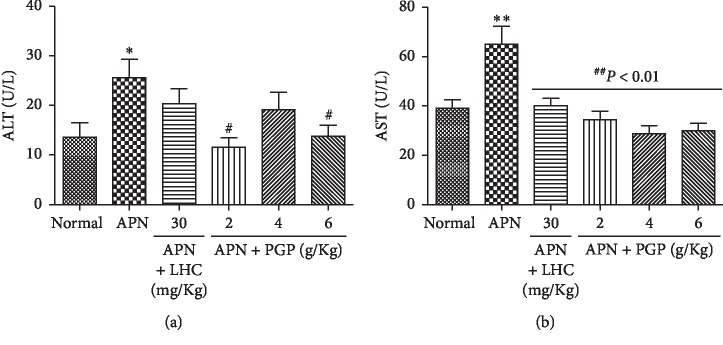
(a) ALT and (b) AST of the APN rat. ^*∗*^*P* < 0.05 compared with the control group; ^*∗∗*^*P* < 0.01 compared with the control group; ^#^*P* < 0.05 compared with the APN group; ^##^*P* < 0.01, compared with the APN group.

AST and ALT levels were significantly increased in the serum of the APN group as compared with the control group (*P* < 0.05 and *P* < 0.01, respectively). The ALT levels of the low-dose and high-dose PGP groups were significantly decreased compared with that of the APN group (*P* < 0.05). The AST levels of the treatment groups were significantly lower than that of the APN group (*P* < 0.01) (Figure 4).

#### 3.8.9. Histopathological Studies

**Figure 5:**
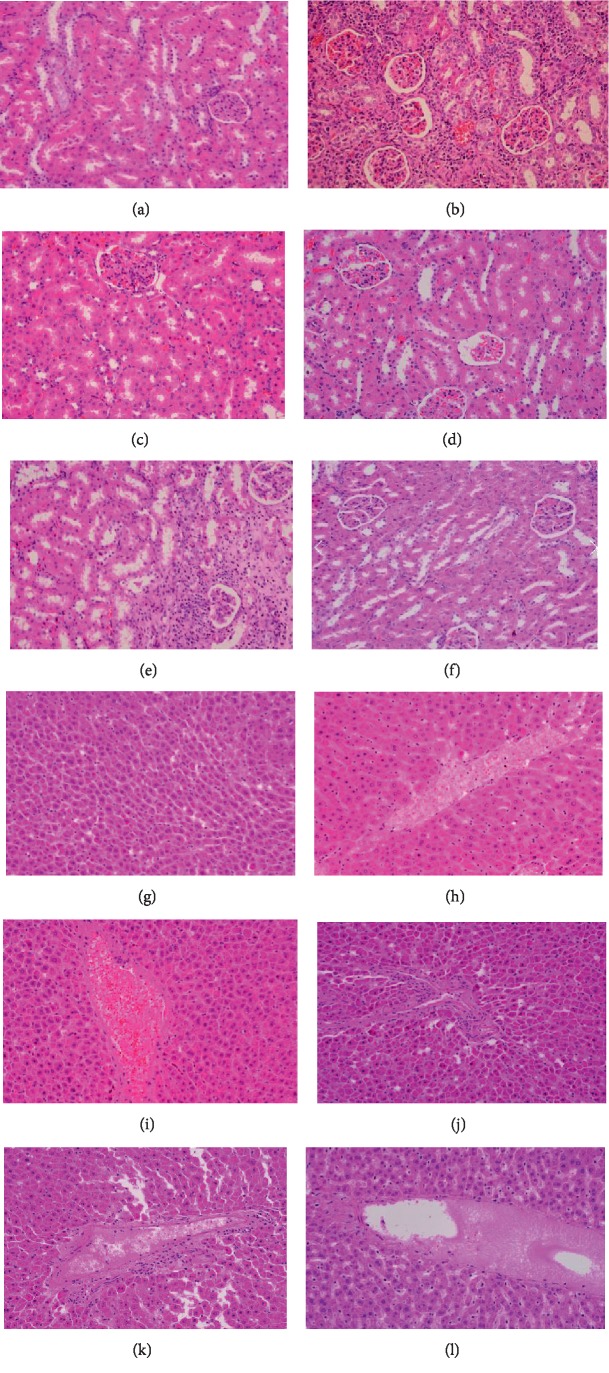
Histologic sections of the kidney (a–f) and the liver (g–l) tissues (400x). (a) control group, (b) APN group, (c) LHC group, (d) low-dose group, (e) middle-dose group, (f) high-dose group, (g) control group, (h) APN group, (i) LHC group, (j) low-dose group, (k) middle-dose group, and (l) high-dose group.

The cross section of the kidney of the APN rat group (Figure 5(b)) shows degeneration of the glomerular with glomerular and tubule interstitial infiltration of lymphocytes, as compared to the control group (Figure 5(a)). In the low-dose group (Figure 5(d)), it appears that APN caused the glomeruli, tubules, and vessels to be congested, which indicates that low-dose PGP has no obvious therapeutic effect. However, better conditions were observed in the middle-dose (Figure 5(e)) and high-dose PGP groups (Figure 5(f)) as compared to the APN group. Compared with the LHC group, certain doses of PGPs have a similar therapeutic effect on APN.

Meanwhile, the cross sections of the liver tissue show that LHC induced obvious hepatotoxicity (Figure 5(i)), and the PGP groups (Figures 5(j)–5(l)) exhibited significant protective action against immunological liver injury in mice (Figure 5). The liver tissue of the APN group was irregular in shape, and the degeneration and necrosis of hepatocytes were severe and the distribution was disordered. In the LHC group, venous congestion occurred in the liver tissue and the liver cells had slight inflammatory infiltration. The liver tissue of the PGP group was normal, with no congestion and inflammation. According to the loss of LHC liver tissue, it may be caused by long-term use of LHC.

### 3.9. Antibacterial Activity of PGPs

The antibacterial activity of PGPs against three Gram-positive bacteria (*M. luteus*, *S. aureus*, and *A. aerogenes*) and one Gram-negative bacteria (*E. coli*) at several concentrations (500 mg/mL, 250 mg/mL, and 125 mg/mL) was investigated. As shown in Table 8, different concentrations of polysaccharides varied in their antibacterial potential, indicating that PGPs have high antibacterial activity.

### 3.10. Diuretic Activity of PGPs

The effect of PGP concentration on diuretic activity was evaluated using standard methods for measurement of urinary output.  Table 9 shows that both PGPs at the higher dose tested (6 g/kg) and standard furosemide (30 mg/kg) significantly (*P* < 0.05) increased the urinary output. Compared with the control group, low-dose PGPs did not generate a diuretic effect (*P* > 0.05). Furthermore, as the concentration of PGPs increased, the urine volume of mice increased significantly. With respect to the total urine volume (diuretic intensity) of each PGP group, the high-dose group had the highest volume, indicating that the high-dose group had the largest urinary output, and thus, the best diuretic effect. There was no difference in the urinary output of the LHC group and the control group. Compared with the LHC group, the PGP groups exhibited a significant increase in the urine output volume, especially in the high-dose PGP group. In conclusion, PGPs appear to have significant diuretic activity, which can last for a long duration of time, whereas LHC has no diuretic effect.

## 4. Discussion

In the present study, the optimum extraction conditions (the decocting time, ratio of water to raw material, and concentration of precipitation ethanol) were determined, with the maximum extraction rate obtained under the lowest cost. Further, the effects of PGPs on APN were investigated in vivo and in vitro through measurement of biochemical indicators of blood and urine, antibacterial activity, antiadhesion activity, and urine output volumes of APN rats and normal mice.

Results indicated that transurethral inoculation with *E. coli* (5 × 10^7^) results in significant nephropyelitis, as evidenced by increased WBC count, NC, bacteria load of the kidney, kidney index, serum creatinine, urine volume, BUN, and IL-2 levels. Treatment with PGPs increased the APN-induced attenuation of sIgA and creatinine clearance and decreased the APN-induced enhancement of WBC count, NC, bacteria load of the kidney, kidney index, serum creatinine, urine volume, BUN, and IL-2 levels. Moreover, we found that intragastric administration of LHC for 14 days caused signs of nephrotoxicity and hepatotoxicity. We also found that treatment of PGPs significantly enhances immunity and decreases urine output volume. Finally, through the diuretic test in normal mice, we demonstrated that administration of PGPs in normal mice for three days is associated with a significant increase in urine output volume. The results of the present study indicate, for the first time, that oral administration of PGPs has a significant, and to some extent dose-dependent, therapeutic effect on *E. coli*-induced APN in rats. In our earlier studies, it was found that PGP had no significant effect on the electrolyte balance in vivo in this work. Ren found that the sodium, potassium, and chloride ion concentrations and osmolality of the rats did not change significantly after receiving PGP [28].

For APN, the antibacterial ability of the urethra and bladder mucous membrane is an important defense mechanism of the body. IL-2 and IgA are important immune indicators in vivo [8, 29, 30]. The spleen is the largest lymphoid organ and is a peripheral immune organ which possesses T and B cells with immunocompetence. Thus, the spleen is an important organ that receives the antigen stimuli and elicits an immune response [31].

An [8] suggested that the promotion of secretion of sIgA from the urethral mucosa can enhance antibacterial and immunity capacity. It has been found that polysaccharides can promote sIgA secretion. In this work, we found that PGP polysaccharide was also consistent with this. IL-2, a Th1-cell-derived cytokine which is involved in cellular immunity, activates human peripheral blood monocytes under several circumstances [32, 33]. Mita et al. [30] showed that IL-2 can upregulate toll-like receptor 4 (TLR4), which plays a role in recognizing Gram-negative bacteria. The spleen index can reflect the immunomodulatory effects of PGPs [29]. After treatment with PGPs, the spleen index and the secretion of sIgA were found to be markedly increased, and this phenomenon has an immunomodulatory function. However, there was a significant decrease in IL-2 concentration, which means there may be a decrease in the expression of TLR4. These results indicate that after treatment with PGPs, immunity is significantly enhanced and the immunomodulatory effect is more remarkable than that induced by LHC treatment.

In addition, it has been reported that APN-induced renal damage is mainly caused by the inflammation process, rather than by the direct effect of bacteria on the kidneys [34–37]. Matsumoto et al. [38] suggested that the inflammatory response following bacterial inoculation is characterized by recruitment of activated neutrophils and lymphocytes to the renal tissue. This phenomenon was evident in the variation in WBC count and neutrophils in our study. The WBC and neutrophil counts were significantly increased in the APN group. Further, the results showed that PGPs can reduce the WBC count and NC to some extent, especially for the high-dose PGP group. We also found that the high-dose PGP group and the LHC group had similar results.

The renal lesions induced by APN, such as tubular atrophy, interstitial inflammation, and interstitial fibrosis, can cause serious damage to renal functions [21]. BUN, UCr, SCr, and ClCr are the most important biochemical indicators of renal function. In this study, the levels of BUN, UCr, SCr, and ClCr were all significantly decreased under PGP treatment. Further, in the high-dose PGP group, all these biochemical indicators were equivalent to the levels seen in the LHC group. Meanwhile, the renal indicators in the low-dose and middle-dose groups were poorer than those of the LHC group. This indicates that the renal function of the rats in the high-dose group was equivalent to the rats in the LHC group; the curative effects of low-dose and middle-dose PGP treatment were not remarkable.

Reducing the bacterial load of the kidneys can alleviate renal damage to some extent. In our study, the microbial counts of the kidneys were significantly reduced after treatment, as compared to the microbial count of the APN group. Even though there was still a difference in the bacterial loads of the PGP and LHC groups, it is clear that PGPs can still reduce the bacterial load of the kidneys to a large extent, especially high-dose PGPs. In addition, our study found that PGPs have no obvious antibacterial activity in vitro whose diameter of the inhibition zone at a concentration of 100 mg/m L was 10.2 mm and the antibacterial activity was weaker than other polysaccharides, such as *Laminaria* polysaccharide [39]. In China, LHC is a frontline broad-spectrum antibiotic, and the in vitro antibacterial activity of LHC was significantly stronger than that of PGPs in the current study.

In order to clarify the mechanisms caused by bacterial pathogens, such as bacterial adhesions, which enable bacteria to reach the kidneys, attach to the epithelial cells, and continue to survive and grow within the urinary tract [26, 40, 41], in the present study, we designed an ex vivo antiadhesion experiment to demonstrate that PGPs can inhibit adhesion to some extent. The effects of high-dose PGPs were better than the other two dosages, but weaker than those observed in the LHC group.

In TCM, PGPs are used as diuretics and as the medicine to alleviate urodynia [9]. In our study, we used normal mice to investigate the diuretic activity of PGPs. Our experimental results showed that the diuretic effects of PGPs were higher than the normal and LHC groups and were markedly higher in the middle-dose and high-dose PGP groups. In other words, LHC does not have a diuretic effect. Importantly, there was no significant difference in the urine volumes of the high-dose group and the furosemide group. Furosemide is a fast and powerful diuretic. We also found that during the first hour, the urine volume of the furosemide group increased rapidly and to a larger extent than that of the high-dose PGP group, but during the final three hours, the urine volume of the furosemide group grew slowly and the urine volume of the high-dose group was larger or equivalent to that of the furosemide group. This indicates that the diuretic effect of PGPs can last longer than the diuretic effect of furosemide.

However, the urine volume of the APN model rats exhibited a different trend. Frequent voiding of small volumes of urine is an important feature of APN. Thus, in our study, the urine volume of the APN group was significantly higher than any other groups. The best effect of decrease in the urine volume was the high-dose PGP group which was better than the effect observed in the LHC group, and the urine volume of the high-dose PGP group was close to the normal group's volume.

In the present study, treatment with PGPs was associated with significantly lower AST and ALT compared to the APN and LHC groups; AST and ALT values in the PGP groups were close to the normal group. The histopathological analysis of liver tissues indicated that the rats in the PGP groups had better liver tissue condition than the LHC group. This phenomenon indicates that bacteria-induced APN can lead to hepatitis. However, our study indicates that LHC has no hepatoprotective effect, while PGPs have a hepatoprotective effect. This characteristic is the advantage that can make PGPs become a potential alternative medicine to treat APN.

It is well established that the most important mechanism of APN is the adhesion effect of bacteria causing the establishment of ascending infection [42]. The mechanism of antiadhesion has been reported. This mechanism is associated with sIgA, which is secreted by the bladder and urethra epithelial cells [8]; sIgA acts as an immune barrier and first-line defense against the invasion of bacteria and is affected by the expression of the polymeric immunoglobulin receptor [43]. Riedash et al. [44]. demonstrated that among women with recurrent UTIs, there was decreased synthesis of sIgA; this decrease in sIgA was regarded as one of the factors that induces UTIs.

Song et al. [45] reported that an innate defense expulsion mechanism defends the uroepithelium from UPEC invasion; this expulsion mechanism depends on TLR4, which is upregulated by IL-2 and is expressed by the uroepithelial cells [8, 42]. In another study, Rice et al. [43] demonstrated that adhesion of PapG, which binds globosides containing glycolipids that are present in the human kidneys, modulates the local secretory antibody immune response by interacting with TLR4 to reduce the expression of polymeric immunoglobulin receptor, thereby decreasing IgA transport through the lamina propria and epithelial cells to the kidney lumen. The authors also suggested that by inhibiting IgA transport into the urinary space, UPEC evades a key host protective mechanism, allowing the ascending infection to establish [43, 46]. This suggests that the mechanism involves the regulation of TLR4 expression by IL-2, which then regulates the content of IgA in the urinary space, preventing the establishment of infection.

Meanwhile, it has been reported that, in vivo, the effect of antiadhesion is related to the scouring effect of urination [8]. Likewise, the diuretic effect of PGPs can improve the scouring effect of urination. Furthermore, it will enhance the antiadhesion ability of PGPs. Thus, PGPs can have the same antiadhesion effect as LHC.

TCM treats APN as a kind of stranguria; the main symptoms of APN are frequent urination and increased nocturia, loose stools, and urodynia [47]. According to the *Compendium of Materia Medica* (*Ben Cao Gang Mu*), PGPs have a diuretic function and can relieve stranguria, alleviate dysentery, and relieve pain. Thus, the diuretic effect of PGPs can not only enhance their antiadhesion ability, but can also promote the excretion of toxins caused by bacteria. In addition, PGPs can markedly ameliorate frequent and urgent urination caused by stranguria (APN). In our study, urine output was found to be decreased in rats in the APN group. The combination of diuresis and relief from stranguria eventually results in the urine volume of APN rats tending to return to normal levels. In TCM, dysentery refers to diarrhea and soft excrement in humans or animals. This is consistent with the phenomenon of loose stools observed in APN rats. In TCM, PGPs are used for treating dysentery, indicating that the effect of PGPs is relative to the disease. Similarly, the pain relief effect of PGPs can also alleviate the problem of urodynia caused by APN, to some extent.

APN is an inflammatory disorder caused by bacterial infection, and relapse occurs easily. It is estimated that 3% of women suffer from very frequent and often constant UTIs [48]. Although antibiotics are the primary treatment protocol for APN at present, there remains a number of treatment challenges, including duration of infection, frequent recurrence, and the increasing risk of resistance to antibiotics. However, it is well known that the majority of antibiotics produce hepatotoxicity. For diseases which are prone to relapse, long-term use of antibiotics can lead to the accumulation of liver toxicity, which may impact on liver function. More importantly, the most problematic aspect of antibiotic use is the increased risk of drug resistance. Nowadays, uropathogen resistance rates to first-line and second-line antibiotic therapies are climbing steadily and multidrug resistance is also on the rise [1]. Schito et al. [49] reported that in 2009, over 10% of cystitis isolates were resistant to at least three different classes of antimicrobial agents. Resistance to antibiotics can complicate treatment and lead to therapeutic failure.

As a TCM with thousands of years of history, the pharmacological effects of peach gum have been recorded in many ancient Chinese works, such as “*Exchange collections of herbal recipes*,” “*Famous physicians record*,” “*Ancient and present recorded recipes*,” “*Compendium of Materia Medica*,” and “*Qian Jin Fang*.” Peach gum also conforms to the idea of medicine food homology. The main components of peach gum are polysaccharides; the content of polysaccharides in peach gum is up to 90%. The polysaccharide content in peach gum is the highest of all known plants, making peach gum a rare and important Chinese medicine resource with high purity [50].

To date, there are no reports suggesting that long-term consumption of PGPs can lead to dependence. Further, according to the results of our study, PGPs exhibit a hepatoprotective effect. Our findings described above suggest that PGPs have a therapeutic effect against APN. PGPs have antiadhesion, diuretic, and stranguria relief functions, which enhance immune regulation, in addition to antibacterial and anti-inflammatory effects (shown in Table 10). These mechanisms are combined to treat APN, and the treatment effect is remarkable. Compared with LHC, PGPs have great advantages in the treatment of APN and stranguria. Our findings indicate that PGPs could become an alternative medicine for APN treatment.

## Figures and Tables

**Table 1:** Independent variables and their levels used in the response surface design.

Independent variable	Unit	Symbol	Low (−1)	Middle (0)	High (1)
Decocting time (*X*_1_)	h	*X*_1_	4	5	6
Ratio of water to raw material (*X*_2_)	mL/g	*X*_2_	80	100	120
Ethanol concentration (*X*_3_)	%	*X*_3_	90	95	100

**Table 2:** RSM experimental design and weighting of the xylose content and extraction yield.

Number	Decocting time	Ratio of water to raw material	Ethanol concentration (%)	Total sugar (%)	Extraction yield (%)	Weighting (%)
1	4	80	95	83.00	70.40	75.44
2	4	120	95	86.25	73.40	78.54
3	4	100	90	82.16	71.60	75.82
4	4	100	100	83.94	77.60	80.14
5	5	120	100	94.38	86.00	89.35
6	5	120	90	93.01	84.60	87.96
7	5	80	100	92.87	82.40	86.59
8	5	80	90	89.21	81.80	84.76
9	6	100	100	91.81	84.40	87.36
10	6	120	95	88.45	85.00	86.38
11	6	100	90	90.62	81.20	84.97
12	6	80	95	89.11	80.60	84.00
13	5	100	95	96.59	87.20	90.96
14	5	100	95	96.30	86.80	90.60
15	5	100	95	96.63	86.40	90.49
16	5	100	95	96.44	86.60	90.54
17	5	100	95	96.68	87.00	90.87

**Table 3:** ANOVA for the quadratic response surface model.

Source	Sum of squares	d*f*	Mean square	*F* value	*P* value	Significance
Model	434.59	9	48.29	195.69	<0.0001	^*∗∗*^
*X*_1_	134.23	1	134.23	543.99	<0.0001	^*∗∗*^
*X*_2_	16.36	1	16.36	66.30	<0.0001	^*∗∗*^
*X*_3_	12.33	1	12.33	49.95	0.0002	^*∗∗*^
*X*_1_ *X*_2_	0.13	1	0.13	0.53	0.4921	
*X*_1_ *X*_3_	0.93	1	0.93	3.77	0.0932	
*X*_2_ *X*_3_	0.048	1	0.048	0.20	0.6712	
*X*_1_^2^	227.29	1	227.29	921.12	<0.0001	^*∗∗*^
*X*_2_^2^	21.41	1	21.41	86.75	<0.0001	^*∗∗*^
*X*_3_^2^	6.82	1	6.82	27.62	0.0012	^*∗∗*^
Residual	1.73	7	0.25			
Lack of fit	1.55	3	0.52	11.76	0.0188	^*∗*^
Pure error	0.18	4	0.044			
Cor total	436.32	16				

*R* ^2^=0.9954, *R*_Adj_^2^=0.9895,  and *R*_Pred_^2^=0.9315. ^*∗*^*P* < 0.05 said the difference was significant and ^*∗∗*^*P* < 0.01 said the difference was significant.

**Table 4:** Linear regression data for the six monosaccharides.

Monosaccharide composition	Standard curve	Linearity range (mg/mL)	Correlation coefficient (*R*^2^)	Monosaccharide concentration (mg/mL)
Mannose	*y* = 6.8661*x* + 1.1978	0.2074–1.8645	0.9927	0.410
Rhamnose	*y* = 6.3682*x* + 1.0041	0.1082–1.3681	0.9946	0.215
Glucose	*y* = 6.2669*x* + 1.0968	0.0522–1.1758	0.9933	0.174
Galactose	*y* = 21.963x − 0.3578	1.2752–3.3763	0.9945	3.452
Xylose	*y* = 16.847x − 0.2507	0.5031–2.2526	0.9983	0.756
Arabinose	*y* = 7.1077*x* + 0.7346	1.1135–5.0133	0.9974	4.993

**Table 5:** Measurement of 24 h urine volume on the 6^th^ day and 12^th^ day (mean ± SD), (*n* = 10).

Group	Normal	APN	LHC	Low-dose PGP	Middle-dose PGP	High-dose PGP
6^th^ day	6.19 ± 1.81	16.75 ± 1.99^*∗∗∗*^	8.34 ± 1.95^###^	14.67 ± 1.31	10.13 ± 0.54^###^	7.10 ± 1.39^###^
12^th^ day	5.23 ± 1.72	13.70 ± 1.86^*∗∗∗*^	9.35 ± 1.91^#^	10.70 ± 1.78	9.25 ± 1.95^#^	7.25 ± 1.75^##^

^*∗∗∗*^ *P* < 0.0001 compared with the control group; ^#^*P* < 0.05 compared with the model group; ^##^*P* < 0.01 compared with the model group; and ^###^*P* < 0.0001 compared with the model group.

**Table 6:** Comparisons of kidney and bladder weights and indices among groups (mean ± SD), (*n* = 10).

Group	Kidney weight	Kidney index	Bladder weight	Bladder index	Spleen weight	Spleen index
Normal	1.69 ± 0.16	0.71 ± 0.07	0.11 ± 0.02	0.05 ± 0.01	0.52 ± 0.05	0.26 ± 0.02
APN	1.96 ± 0.15^*∗∗*^	0.82 ± 0.06^*∗∗∗*^	0.22 ± 0.07^*∗∗∗*^	0.09 ± 0.03^*∗∗∗*^	0.65 ± 0.12^*∗∗*^	0.22 ± 0.01^*∗∗*^
LHC	1.77 ± 0.10^##^	0.74 ± 0.04^##^	0.17 ± 0.04^#^	0.07 ± 0.02^#^	0.56 ± 0.04	0.23 ± 0.02^*∗*^
Low-dose PGP	1.74 ± 0.10^##^	0.73 ± 0.04^#^	0.20 ± 0.04	0.09 ± 0.01	0.67 ± 0.06^###^	0.28 ± 0.02^###^
Middle-dose PGP	1.84 ± 0.19	0.77 ± 0.08	0.25 ± 0.05	0.11 ± 0.02	0.56 ± 0.14^#^	0.27 ± 0.03^##^
High-dose PGP	1.65 ± 0.29^#^	0.69 ± 0.12^###^	0.15 ± 0.05^##^	0.06 ± 0.02^##^	0.51 ± 0.07	0.23 ± 0.03

^*∗*^ *P* < 0.05 compared with the control group; ^*∗∗*^*P* < 0.01 compared with the control group; ^*∗∗∗*^*P* < 0.001 compared with the control group; ^#^*P* < 0.05 compared with the model group; ^##^*P* < 0.01 compared with the model group; and ^###^*P* < 0.001 compared with the model group.

**Table 7:** Levels of BUN, SCr, UCr, ClCr, and CRP (mean ± SD), (*n* = 10).

Group	BUN (mmol·L^−1^)	SCr (*μ*mol·L^−1^)	UCr	ClCr	CRP (mg/L)
Normal	7.63 ± 0.26	301.26 ± 7.95	1504.84 ± 269.28	1.68 ± 0.73	0.55 + 0.12
APN	9.25 ± 0.80^*∗*^	327.78 ± 24.47^*∗*^	2751.52 ± 724.81^*∗∗*^	0.55 ± 0.25^*∗∗*^	1.28 + 0.21^*∗∗*^
LHC	8.27 ± 1.08	298.99 ± 15.36^#^	2235.03 ± 311.38	0.93 ± 0.42^#^	0.67 + 0.26^###^
Low-dose PGP	5.26 ± 0.75^###^	293.88 ± 20.33^#^	2527.13 ± 357.95	1.85 ± 0.26^###^	1.12 + 0.33^#^
Middle-dose PGP	6.03 ± 1.40^###^	326.80 ± 14.95	1445.40 ± 270.84^###^	0.62 ± 0.21	0.72 + 0.215^##^
High-dose PGP	6.49 ± 1.51^###^	282.80 ± 16.40^##^	1858.87 ± 789.15^#^	1.04 ± 0.44^#^	0.58 + 0.51^###^

^*∗*^ *P* < 0.05 compared with the control group; ^*∗∗*^*P* < 0.01 compared with the control group; ^#^*P* < 0.05 compared with the model group; ^##^*P* < 0.01 compared with the model group; and ^##^*P* < 0.001 compared with the model group.

**Table 8:** Diameter of the inhibition zone (mm) (mean ± SD), (*n* = 10).

Group	Antibacterial circle diameter (mm)			
	*E. coli*	*M. luteus*	*S. aureus*	*A. aerogenes*
PGP (500 mg/mL)	15.5 ± 0.33	13.5 ± 0.28	18.0 ± 0.19	16.5 ± 0.23
PGP (250 mg/mL)	10.0 ± 0.24	9.6 ± 0.17	13.0 ± 0.38	11.5 ± 0.22
PGP (125 mg/mL)	8.5 ± 0.32	8.0 ± 0.26	8.6 ± 0.29	8.0 ± 0.32
Sterile water	7.8 ± 0.00	7.8 ± 0.00	7.8 ± 0.00	7.8 ± 0.00

**Table 9:** Effect of 3-day oral dose of peach gum polysaccharide solution (PGPS) and furosemide on urinary volume (4 h) (mean ± SD) (*n* = 5).

Group	Urine volume (mL/100 g body weight)				
	0-1 h	1-2 h	2-3 h	3-4 h	Diuretic intensity (%)
Control	1.44 ± 0.19	1.12 ± 0.20	1.18 ± 0.28	1.16 ± 0.20	100.0
Furosemide	7.78 ± 0.32^*∗∗∗*^	3.32 ± 0.28^*∗∗∗*^	1.48 ± 0.36	1.32 ± 0.32	261.9
LHC	2.12 ± 0.40	1.48 ± 0.43	1.36 ± 0.24	1.24 ± 0.16	115.3
PGP (2 g/kg)	2.95 ± 0.45^*∗*^	1.88 ± 0.16^*∗∗*^	1.60 ± 0.28	1.28 ± 0.16	139.6
PGP (4 g/kg)	3.08 ± 0.16^*∗∗∗##*^	2.88 ± 0.40^*∗∗∗##*^	1.96 ± 0.60	1.36 ± 0.24	172.4
PGP (6 g/kg)	4.32 ± 0.42^*∗∗∗###*^	4.12 ± 0.12^*∗∗∗###*^	2.08 ± 0.45^*∗*^	2.12 ± 0.20^*∗∗∗###*^	224.6

^*∗*^ *P* < 0.05 compared with the control group; ^*∗∗*^*P* < 0.01 compared with the control group; ^*∗∗∗*^*P* < 0.001 compared with the control group; ^##^*P* < 0.01 compared with the LHC group; and ^###^*P* < 0.001 compared with the LHC group.

**Table 10:** The result of comparison of efficacy evaluation indicators.

Efficacy evaluation indicators	Antibacterial	Diuretic	Kidney protection					Liver protection			Antiadhesion		Immune regulation		Anti-inflammatory	
	Antibacterial	Urine volume of 4 h	BUN	SCr	UCr	ClCr	Histopathology	ALT	AST	Histopathology	Microbial counts in kidney	Hemagglutination assay	sIgA	WBC	IL-2	NC
APN		↑↑↑	↑	↑	↑↑	↑↑	↑↑	↑	↑↑	↑↑↑	↑↑↑		↑↑↑	↑↑	↑↑	↑
LHC		↓	↓	↓		↓	↓		↓↓	↓	↓↓↓	↑↑↑			↓	↓↓↓
Low-dose PGP	↑		↓	↓		↓↓↓	↓	↓	↓↓	↓	↓↓↓	↑			↓	
Middle-dose PGP	↑↑	↓	↓↓↓		↓↓↓		↓		↓↓	↓	↓↓↓	↑↑			↓	
High-dose PGP	↑↑↑	↓↓	↓↓↓	↓↓	↓	↓	↓	↓	↓↓	↓↓	↓↓↓	↑↑↑	↓↓↓	↓↓↓	↓	↓

↑, increase; ↓, decrease. ^↑^*P* < 0.05 compared with the control group; ^*⇈*^*P* < 0.01 compared with the control group; ^*↑↑↑*^*P* < 0.001 compared with the control group; ^↑^*P* < 0.05 compared with the model group; ^*⇈*^*P* < 0.01 compared with the model group; ^*↑↑↑*^*P* < 0.001 compared with the model group.

## Data Availability

All data generated or analyzed during this study are included in this article.

## Abbreviations

*A. aerogenes*: *Aerobacter aerogenes*
ALT: Alanine aminotransferase
APN: Acute pyelonephritis
AST: Aspartate transaminase
BUN: Blood urea nitrogen
ClCr: Creatinine clearance
*E. coli*: *Escherichia coli*
IL-2: Interleukin-2
LHC: Levofloxacin hydrochloride capsules
*M. luteus*: *Micrococcus luteus*
NC: Neutrophil count
PGP: Peach gum polysaccharide
*S. aureus*: *Staphylococcus aureus*
SCr: Serum creatinine
sIgA: Secretory immunoglobulin A
UCr: Urine creatinine
WBC: White blood cells.

## Supplementary Materials

Supplementary Materials The concise description of the graphic abstract. Peach gum has long been recognized by traditional Chinese medicine as a food with medicinal value of relieving stranguria, could be used for cure of acute pyelonephritis (APN), but whether and how its primary constituent peach gum polysaccharides (PGPs) contribute to the diuretic function is still not clear. In this study, surface design optimization was adopted to optimize the preparation of PGPs, and HPLC and FT-IR spectra were used to evaluate the quality of PGPs; APN model rat was established by *Escherichia coli* urinary tract infection method; the therapeutic effect and mechanism of PGPs on APN were determined by the visceral index, biochemical indicators, pathological section of the APN rat, and diuretic activity on mice and antibacterial activity in vitro. The results showed that treatment with PGPs increased the APN-induced attenuation of secretory immunoglobulin A (sIgA) and creatinine clearance, and decreased the APN-induced enhancement of the number of white blood cell (WBC), neutrophil counts (NC), bacteria load of the kidneys, kidney index, serum creatinine, urine volume, blood urea nitrogen (BUN), and interleukin-2 (IL-2) levels. The mechanism underlying these effects was further elucidated through in vitro experiments of the antibacterial and antiadhesion effects of PGPs.
