# Supplementary figures and images for: Investigation of the Optimum Preparation of Peach Gum Polysaccharides and the In Vivo and In Vitro Therapeutic Effects on Acute Pyelonephritis
Source: Evid Based Complement Alternat Med. 2019 Dec 12;2019:2729343. doi: 10.1155/2019/2729343 (PMC6930766; doi:10.1155/2019/2729343)

**
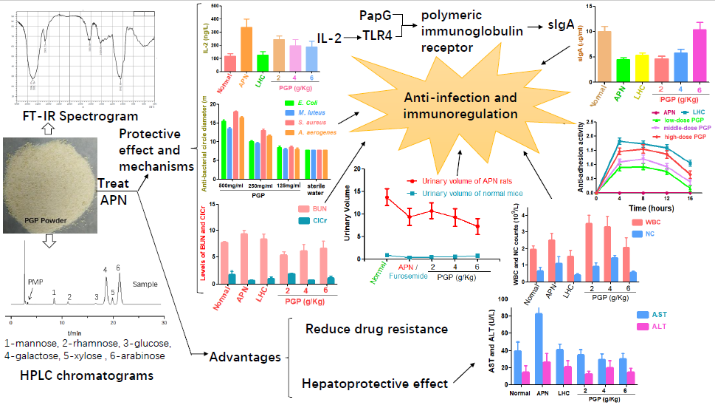
**

Supplement: Supplementary Materials — The concise description of the graphic abstract. Peach gum has long been recognized by traditional Chinese medicine as a food with medicinal value of relieving stranguria, could be used for cure of acute pyelonephritis (APN), but whether and how its primary constituent peach gum polysaccharides (PGPs) contribute to the diuretic function is still not clear. In this study, surface design optimization was adopted to optimize the preparation of PGPs, and HPLC and FT-IR spectra were used to evaluate the quality of PGPs; APN model rat was established by Escherichia coli urinary tract infection method; the therapeutic effect and mechanism of PGPs on APN were determined by the visceral index, biochemical indicators, pathological section of the APN rat, and diuretic activity on mice and antibacterial activity in vitro. The results showed that treatment with PGPs increased the APN-induced attenuation of secretory immunoglobulin A (sIgA) and creatinine clearance, and decreased the APN-induced enhancement of the number of white blood cell (WBC), neutrophil counts (NC), bacteria load of the kidneys, kidney index, serum creatinine, urine volume, blood urea nitrogen (BUN), and interleukin-2 (IL-2) levels. The mechanism underlying these effects was further elucidated through in vitro experiments of the antibacterial and antiadhesion effects of PGPs. [file 2729343.f1.docx]
